# Experiential Classes Plus Digital Logging in Antenatal Care for Pregnant Women in China: Mixed Methods Study

**DOI:** 10.2196/84705

**Published:** 2026-04-21

**Authors:** Zhenfeng Sun, Fuwen Yang, Xi Wang, Yin Sun, Suhan Zhang, Liangkun Ma

**Affiliations:** 1 Department of Maternal Health Care, Beijing Daxing District Maternal and Child Health Hospital, Beijing, China; 2 School of Social Sciences, Faculty of Arts and Education, University of Auckland, Auckland, New Zealand; 3 School of Community and Public Health, Faculty of Health and Environment Sciences, Auckland University of Technology, Auckland, New Zealand; 4 Department of Obstetrics and Gynecology, National Clinical Research Center for Obstetric & Gynecologic Diseases, Peking Union Medical College Hospital, Chinese Academy of Medical Sciences & Peking Union Medical College, No. 1 Shuaifuyuan, Dongcheng District, Beijing, 100730, China, 86 13021961166

**Keywords:** maternal health, antenatal education, experiential learning, digital health, mixed methods, China

## Abstract

**Background:**

Maternal health during the perinatal period is a global public health priority. While antenatal education is widely implemented, conventional lecture-based models often fail to achieve sustained behavior change. Innovative approaches that integrate experiential learning with digital support may enhance maternal knowledge, self-management, and pregnancy outcomes.

**Objective:**

The aim of this study is to evaluate the feasibility and preliminary effectiveness of a combined experiential class and online logging intervention for pregnant women in China and to explore the mechanisms underpinning its impact on health practices and service experiences.

**Methods:**

A mixed methods design was used in a district-level maternal and child health hospital in Beijing. In the quantitative arm, 40 women (intervention group, n=20; control group, n=20) were enrolled in a quasi-experimental comparison. Outcomes included knowledge-attitude-practice indicators, service satisfaction, and clinical birth outcomes. Given the limited sample size, a qualitative arm was conducted to complement statistical findings: semistructured interviews with 20 women (10 per group) were analyzed thematically. Quantitative and qualitative results were integrated during interpretation to provide a comprehensive evaluation.

**Results:**

Compared with the experiential class alone, the combined intervention was associated with higher knowledge scores (mean difference 1.6 points, 95% CI 0.8-2.4), stronger adherence to recommended health practices (composite adherence score difference 1.0, 95% CI 0.4-1.6), and higher overall service satisfaction (mean difference 0.6, 95% CI 0.2-1.0). Across multiple domains, a higher proportion of participants in the intervention group met dietary, exercise, and supplementation recommendations. Clinical outcome differences were exploratory, as the study was not powered for these end points. Qualitative analysis revealed 3 mechanisms, such as empowerment and self-efficacy, practice and persistence, and systemic/environmental support, through which the intervention influenced experiences and practices.

**Conclusions:**

The experiential class plus online logging model is feasible and acceptable in a real-world antenatal setting. Although limited by a small sample size, findings suggest that the intervention improves maternal knowledge, health practices, and service experiences and may inform future adequately powered trials to evaluate pregnancy outcomes. Qualitative insights highlight mechanisms of health practice change and provide contextual depth, underscoring the value of mixed methods designs in maternal health research.

## Introduction

Maternal health during the perinatal period remains a major global public health priority. Despite significant progress in reducing maternal mortality worldwide, complications, such as gestational diabetes mellitus, hypertensive disorders of pregnancy, and anemia, continue to contribute substantially to adverse maternal and neonatal outcomes [1,2]. The World Health Organization (WHO) emphasizes that most maternal morbidity and mortality are preventable through timely interventions, health promotion, and improved access to high-quality care [3]. In China, the Healthy China 2030 strategy underscores maternal and child health as a key domain, with specific targets for improving antenatal care (ANC) coverage, optimizing service quality, and reducing complications [4,5]. However, changing sociodemographic trends, including advanced maternal age, increased prevalence of obesity, and sedentary lifestyles, have exacerbated risks and raised new challenges for maternal health promotion [6,7].

The perinatal period represents both a vulnerable and transformative stage. Physiological and psychological changes heighten women’s susceptibility to stress, anxiety, and health complications, while simultaneously creating a heightened motivation to adopt healthier behaviors [8,9]. Evidence demonstrates that maternal lifestyle behaviors, including balanced nutrition, regular exercise, appropriate weight management, and adherence to medical guidance, are critical determinants of pregnancy outcomes and long-term maternal and child health trajectories [10,11]. Nevertheless, translating knowledge into sustained practices is not straightforward. Pregnant women frequently report difficulties in maintaining dietary modifications, establishing exercise routines, and managing stress, even when aware of recommended guidelines [12]. This “knowledge–practice gap” highlights the limitations of existing antenatal education models in bridging intention with behavior.

In China, antenatal education is widely available but often delivered in a didactic, lecture-based format within hospital settings. While such programs increase awareness, they frequently fail to address the complexity of behavior change, the need for continuous reinforcement, or the psychosocial dimensions of pregnancy [13,14]. Such didactic communications, positioning women as passive recipients rather than active participants, lack interactivity and may, thus, reduce motivation, undermine confidence, and leave critical needs unmet, such as emotional reassurance, social connection, and patient–provider trust [15]. To mitigate this gap in antenatal education, experiential and participatory learning approaches have been engaged and increasingly recognized as promising strategies to ensure that antenatal education is practical, engaging, and responsive to the lived experiences of pregnant women. Unlike traditional lectures, experiential classes emphasize active engagement through demonstration, simulation, and discussion [16,17], helping women translate abstract knowledge into practical skills and, in turn, enhancing comprehension, self-efficacy, and readiness for behavior change [18,19]. It should also be noted that experiential teaching, as a pedagogical approach, may entail an intention-behavior gap [20], which could be magnified by the complex external environments and practical barriers characteristic of pregnancy [21,22]. At the same time, digital health interventions, including mobile applications, online tracking tools, and peer support platforms, offer continuity of care beyond the clinic, providing real-time monitoring, feedback, and social reinforcement [23,24]. In China, the widespread adoption of platforms, such as WeChat, creates opportunities for embedding low-cost, scalable, and culturally integrated digital health solutions into routine ANC [25].

Although both experiential education and digital interventions have demonstrated potential independently, limited research has integrated these approaches into a combined model and evaluated their effects in real-world antenatal settings. Moreover, most existing evaluations rely predominantly on quantitative outcomes, leaving the mechanisms through which such interventions influence maternal engagement and health behaviors underexplored [26,27]. This study addresses these gaps by piloting an experiential class reinforced with a 7-day online logging program in a district-level maternal and child health hospital in Beijing, designed to test whether digital reinforcement can enhance the pedagogical effects of experiential learning, particularly by strengthening maternal self-efficacy and translating readiness into actual practice changes. A mixed methods design was adopted to capture both measurable outcomes and explanatory insights. The quantitative component assessed changes in knowledge, health practices, satisfaction, and pregnancy outcomes between intervention and control groups, whereas the qualitative component explored participants’ lived experiences and explanatory mechanisms of change. Accordingly, the aim of this study was to evaluate the feasibility and preliminary effectiveness of this combined intervention, with particular attention to its impact on maternal self-efficacy and health practices, and to generate insights to inform the design of longer-term, scalable antenatal education programs.

## Methods

### Study Design

This study employed a convergent mixed methods design [28], combining a quasi-experimental quantitative comparison with qualitative inquiry. The quantitative arm assessed changes in maternal knowledge, health practices, satisfaction, and pregnancy outcomes between intervention and control groups. Recognizing the limitations of a relatively small sample size, the qualitative arm was designed to complement quantitative findings, provide explanatory depth, and explore mechanisms of practice change. Quantitative and qualitative results were analyzed separately and integrated during the interpretation stage to enable a comprehensive evaluation.

### Setting and Participants

The study was conducted between January and December 2024 in a district-level maternal and child health hospital in Beijing. Women attending routine ANC were invited to participate in the quantitative arm. Eligibility criteria included singleton pregnancy (24‐34 wk gestation), ability to attend antenatal classes, access to a smartphone for WeChat use, and provision of informed consent. Exclusion criteria included severe pregnancy complications (eg, preeclampsia, placenta previa requiring hospitalization), preexisting metabolic or cardiovascular conditions, and cognitive or communication difficulties. A total of 40 women were enrolled, with 20 attending an experiential antenatal class only (control group) and 20 attending the same class followed by a 7-day online logging program (intervention group). As part of routine study management, participants in each arm were also connected via a closed WeChat group. The qualitative arm was nested within this cohort. Twenty women, drawn purposively from both groups to ensure variation in age, parity, and engagement levels, were invited for in-depth interviews (10 intervention and 10 control). Participants were anonymized using alphanumeric codes (DK01-DK10 for intervention; WDK01-WDK10 for control).

### Intervention

**Figure 1.**
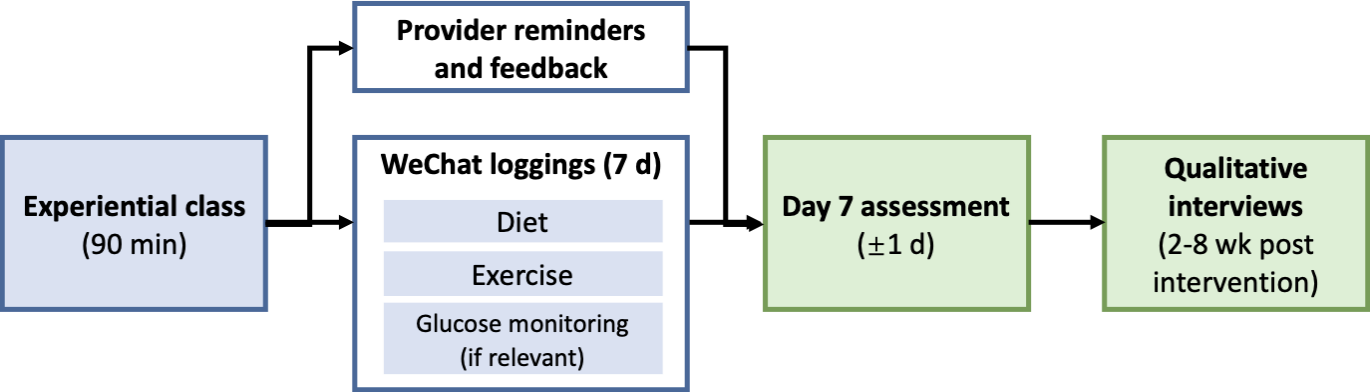
Intervention workflow. All participants attended a 90-minute experiential antenatal class. The intervention group additionally used a WeChat miniprogram for 7 consecutive days to log diet, exercise, and (where relevant) glucose monitoring, supported by provider reminders and WeChat-based messaging feedback. Outcomes were assessed on day 7 (±1 d) in both groups; qualitative interviews were conducted 2 to 8 weeks post intervention.

The intervention comprised 2 components. First, all participants attended a 90-minute session delivered by obstetricians, midwives, and nutritionists. This session combined lecture-style teaching with demonstration, role-play, group discussions, and practice exercises, and covered key topics, such as pregnancy nutrition, gestational weight management, prevention of gestational diabetes and anemia, and safe physical activity. The control group received this standardized experiential class only. The intervention group was enrolled in a WeChat-based miniprogram for 7 consecutive days, during which they were asked to log daily information on diet; exercise; and, where relevant, blood glucose monitoring. Health care providers monitored entries, provided reminders, and offered personalized feedback through the miniprogram, ensuring continuity of support beyond the classroom. The overall intervention workflow is shown in Figure 1. Thus, the 2 groups differed only in the presence of the digital logging component.

In addition, separate closed WeChat groups were established for the intervention and control arms to facilitate routine communication, study management, and responses to participants’ questions. Group access was restricted to enrolled participants and required manual approval by the group administrator (a research team member; the same administrator managed both groups). There was no fixed discussion theme; however, participants were informed that irrelevant content or commercial advertisements were not permitted. The study team proactively posted logging reminders and the miniprogram entry link in the intervention group only, whereas the control group did not receive proactive messages. In both groups, staff responded to questions raised by participants. Personalized feedback on the logged content was delivered to individual participants within the miniprogram rather than posted in the group chat.

### Data Collection

Outcomes assessed corresponded to the quantitative result domains that include maternal knowledge, health practices, service experience, and selected clinical outcomes. Knowledge, health practices, and service experience were assessed once at a standardized 7-day postclass follow-up for both groups. During the 7-day period, participants in the intervention arm logged diet, exercise, and (where applicable) glucose readings via a WeChat miniprogram with provider feedback, whereas controls received the class without any digital component. A structured knowledge–attitude–practice questionnaire [29] was administered on day 7 (±1 d). The knowledge section comprised 7 items on pregnancy nutrition, weight management, gestational diabetes mellitus prevention, and anemia recognition, yielding a total score of 0 to 7 (1 point per correct response). Health practice indicators were coded as binary according to prespecified thresholds: dietary management ≥5 days/week, physical activity ≥3 times/week, regular supplement use (daily or every other day), and sharing health information with family (occasionally/often). Service experience was measured on a 5-point Likert scale (clarity of communication, respect for needs, personalization, and overall satisfaction). Clinical outcomes (labor duration, delivery mode, perineal trauma, postpartum hemorrhage, gestational complications, and neonatal outcomes such as birth weight, asphyxia, and malformations) were abstracted from medical records.

For the qualitative data, semistructured interviews were conducted 2 to 8 weeks after the intervention. The interview guide explored (1) emotional responses to the program, (2) perceived changes in knowledge and behavior, (3) experiences of peer and provider interaction, and (4) views on the applicability of learned strategies in daily life. Interviews lasted 10 to 25 minutes, were audio-recorded, transcribed verbatim, and anonymized prior to analysis.

### Data Analysis

Quantitative data were analyzed using SPSS version 25.0 (IBM Corp). Descriptive statistics were used to summarize baseline characteristics and outcome measures, with continuous variables presented as means and SDs and categorical variables expressed as frequencies and percentages. Group comparisons were explored using *χ*^2^ or Fisher exact tests for categorical data and independent-samples *t* tests for continuous data where appropriate. However, given the modest sample size, emphasis was placed on reporting effect sizes and 95% CIs to indicate the magnitude [30] and direction of differences rather than relying solely on *P* values for statistical significance. All quantitative findings were interpreted as preliminary trends and considered alongside qualitative results to provide a more comprehensive understanding of the intervention’s impact.

Qualitative data from semistructured interviews were analyzed thematically following the Braun and Clarke 6-step approach [31]. Transcripts were read repeatedly for familiarization, coded independently by 2 researchers, and iteratively developed into themes through discussion and refinement. Discrepancies were resolved by consensus, and representative quotations were selected to illustrate key points. Rigor was enhanced through member checking with a subset of participants and triangulation with class records and log data. Integration of quantitative and qualitative findings occurred at the interpretation stage: while the quantitative results identified patterns of change in knowledge, behaviors, and outcomes, the qualitative analysis provided insight into the mechanisms through which these changes occurred, thereby adding contextual depth and explanatory power.

Integration of findings was conducted at the interpretation stage using a convergent mixed methods approach. Quantitative results identified measurable changes in knowledge, behaviors, satisfaction, and selected clinical outcomes, whereas qualitative narratives offered insights into the processes and mechanisms underlying these changes. The 2 strands were brought together to assess complementarity, convergence, and divergence: quantitative trends pointing to improved dietary adherence, supplement use, and self-monitoring were supported by qualitative accounts of enhanced confidence, emotional reassurance, and peer support; observed differences in maternal outcomes were contextualized by participants’ descriptions of how new practices were adopted and sustained. In this way, the integration of datasets provided a more comprehensive understanding of both the effects and the pathways of the intervention than either method could yield alone.

To ensure the validity and trustworthiness of the findings, several strategies were adopted across both components of the study. For the quantitative analysis, descriptive statistics and effect sizes were reported in addition to *P* values, with 95% CIs used to illustrate the precision of estimates. Given the limited sample size, findings were interpreted cautiously as preliminary trends rather than definitive effects. For the qualitative component, rigor was enhanced through independent dual coding, iterative theme development, and resolution of discrepancies by consensus. Member checking was undertaken with a subset of participants to confirm the resonance of interpretations with their lived experiences, and triangulation with classroom records and digital log data was applied to strengthen confirmability. Reflexive notes and coding logs were maintained to document analytic decisions, ensuring transparency and dependability. Finally, the integration of quantitative and qualitative findings followed a structured convergent mixed methods framework, which enabled the systematic comparison of results and enhanced the overall credibility of conclusions by drawing on multiple sources of evidence.

### Ethical Considerations

The study protocol was reviewed and approved by the Ethics Committee of Beijing Daxing District Maternal and Child Health Hospital (approval number: LLSC2024014). All participants provided written informed consent prior to participation. Participants did not receive any financial compensation, reimbursement, or other incentives for participation. The study complied with the principles of the Declaration of Helsinki. Data confidentiality was safeguarded through anonymization and the secure storage of both quantitative and qualitative materials.

For the digital component, the WeChat miniprogram collected only study-relevant behavioral logs (diet, exercise, and glucose values, where applicable) and did not require participants to enter national identification numbers or unrelated personal data. Access to the administrative interface was restricted to authorized study staff via password-protected accounts. Exported datasets were deidentified prior to analysis and stored on encrypted, access-controlled institutional computers. Any participant quotations used in reporting were anonymized.

As communication also occurred within closed WeChat groups, residual privacy risks inherent to social messaging environments could not be fully eliminated, including participant-initiated screenshots or secondary sharing beyond the research team’s control. To mitigate this, group membership was restricted to enrolled participants with manual administrator approval, and participants were informed at enrollment not to disclose identifiable or highly personalized information in the group chat.

## Results

### Quantitative Results

A total of 40 pregnant women completed the quantitative component of the study, with 20 assigned to the intervention group (experiential class plus online logging) and 20 to the control group (experiential class only).

#### Baseline Characteristics

Baseline demographic and obstetric characteristics were generally compared between groups (Table 1). The mean maternal age was 33.6 (SD 3.9) years in the intervention group and 31.8 (SD 4.6) years in the control group. Gravidity and parity were similar across groups (mean 1.75, SD 1.07 vs mean 1.85, SD 1.04; mean 1.4, SD 0.60 vs mean 1.3, SD 0.66). Mean prepregnancy BMI was 21.9 (SD 1.6) in the intervention group and 23.9 (SD 4.2) in the control group, with mean corresponding prepregnancy weights of 58.0 (SD 4.8) kg and 62.7 (SD 9.9) kg. Height was also comparable (mean 162.2, SD 6.1 cm vs mean 161.3, SD 6.0 cm). The proportion of primiparas was slightly lower in the intervention group (26/40, 65% vs 32/40, 80%). Mean gestational age at delivery was 39.1 (SD 1.0) weeks in the intervention group and 39.1 (SD 1.2) weeks in the control group. No statistically significant differences were observed between groups at baseline.

**Table 1.** Baseline characteristics of participants (N=40).

Characteristic	Intervention (n=20)	Control (n=20)
Age (y), mean (SD)	33.6 (3.9)	31.8 (4.6)
Gravidity, mean (SD)	1.75 (1.07)	1.85 (1.04)
Parity, mean (SD)	1.4 (0.60)	1.3 (0.66)
Height (cm), mean (SD)	162.2 (6.1)	161.3 (6.0)
Prepregnancy weight (kg), mean (SD)	58.0 (4.8)	62.7 (9.9)
Prepregnancy BMI, mean (SD)	21.9 (1.6)	23.9 (4.2)
Primiparity, n (%)	13 (65)	16 (80)
Gestational age at delivery (wk), mean (SD)	39.1 (1.0)	39.1 (1.2)

#### Incremental Effects of Online Logging on Maternal Knowledge, Health Practices, and Satisfaction

At the postintervention assessment, women in the intervention group demonstrated significantly higher knowledge scores, stronger adherence to healthy practices, and greater satisfaction with services compared with the control group (Table 2).

The mean knowledge score was 6.8 (SD 0.9) in the intervention group and 5.2 (SD 1.1) in the control group, corresponding to a mean difference of 1.6 points (95% CI 0.8-2.4). Correct response rates for specific domains were also consistently higher: weight management (18/20, 90% vs 13/20, 65%; risk difference 25%, 95% CI 4%-46%), gestational diabetes prevention (19/20, 95% vs 12/20, 60%; risk difference 35%, 95% CI 15%-55%), and anemia recognition (16/20, 80% vs 11/20, 55%; risk difference 25%, 95% CI 3%-47%).

Intervention participants also showed stronger adherence to recommended practices. The mean composite adherence score was 3.3 (SD 0.7) in the intervention group compared with 2.3 (SD 0.9) in the control group (mean difference 1.0, 95% CI 0.4-1.6). Dietary adherence (≥5 days per week) was achieved by 17 out of 20 (85%) intervention participants versus 12 out of 20 (60%) controls (risk difference 25%, 95% CI 2%-47%). Similarly, 16 out of 20 (80%) intervention participants engaged in regular exercise versus 11 out of 20 (55%) controls (risk difference 25%, 95% CI 3%-47%), and 17 out of 20 (85%) intervention participants reported consistent supplement use versus 10 out of 20 (50%) controls (risk difference 35%, 95% CI 12%-58%). Knowledge sharing with family members was also more common (16/20, 80% vs 13/20, 65%), although the risk difference (15%, 95% CI –8% to 38%) included 0, suggesting that this trend was not statistically robust.

**Table 2.** Maternal knowledge, behavior adherence, and service satisfaction between groups at postintervention assessment.

Indicator	Intervention (n=20)	Control (n=20)	Mean/risk difference (95% CI)
Mean knowledge score (0‐7), mean (SD)	6.8 (0.9)	5.2 (1.1)	1.6 (0.8 to 2.4)
Weight management knowledge correct, %	90	65	25 (4 to 46)
GDM^a^ prevention knowledge correct, %	95	60	35 (15 to 55)
Anemia recognition correct, %	80	55	25 (3 to 47)
Composite behavior score (0‐4), mean (SD)	3.3 (0.7)	2.3 (0.9)	1.0 (0.4 to 1.6)
Dietary adherence (≥5 d/wk), %	85	60	25 (2 to 47)
Exercise adherence (≥3 times/wk), %	80	55	25 (3 to 47)
Regular supplement use, %	85	50	35 (12 to 58)
Health knowledge sharing with family, %	80	65	15 (–8 to 38)
Overall satisfaction score (1-5), mean (SD)	4.6 (0.5)	4.0 (0.6)	0.6 (0.2 to 1.0)
Clarity of provider communication, %	100	90	10 (–5 to 25)
Respect for personal needs, %	100	85	15 (–1 to 31)
Receipt of personalized advice, %	95	70	25 (6 to 44)
Overall satisfaction, %	95	80	15 (–4 to 34)

a GDM: gestational diabetes mellitus.

Service experience outcomes also favored the intervention group. Overall satisfaction scores were higher (mean 4.6, SD 0.5 vs mean 4.0, SD 0.6; mean difference 0.6, 95% CI 0.2-1.0). A higher proportion of intervention participants reported clear communication (20/20, 100% vs 18/20, 90%; risk difference 10%, 95% CI –5% to 25%) and respectful care (20/20, 100% vs 17/20, 85%; risk difference 15%, 95% CI –1% to 31%), though the 95% CI included 0. Receipt of personalized advice (19/20, 95% vs 14/20, 70%; risk difference 25%, 95% CI 6%-44%) was consistently higher in the intervention group. Overall service satisfaction reached 19 out of 20 (95%) in the intervention group compared with 16 out of 20 (80%) in controls (risk difference 15%, 95% CI –4% to 34%).

Across all knowledge, health practices, and service experience indicators, mean values and proportions in the control group were consistently lower than those in the intervention group, indicating a general trend favoring the addition of online logging, although some confidence intervals crossed 0.

#### Effect on Maternal and Neonatal Outcomes

Clinical outcomes were exploratory. Given the pilot sample size, the study was not powered to detect differences in maternal or neonatal end points; therefore, between-group differences should be interpreted as preliminary and hypothesis-generating rather than evidence of clinical benefits (Table 3). Mean labor duration was 7 hours 36 minutes (SD 247 min) in the intervention group compared with 8 hours 11 minutes (SD 255 min) among controls. Postpartum blood loss was lower in the intervention group (mean 398, SD 105 ml vs mean 447, SD 138 ml). Moderate-to-severe perineal trauma occurred in 8 out of 20 (40%) intervention participants versus 10 out of 20 (50%) controls. No clear differences were observed for cesarean section rates (10/20, 50% vs 9/20, 45%).

**Table 3.** Maternal and neonatal outcomes.

Outcome	Intervention (n=20)	Control (n=20)
Weight gain (kg), mean (SD)	12.2 (4.8)	11.3 (3.7)
Postpartum blood loss (ml), mean (SD)	398 (105)	447 (138)
Labor duration, mean (SD)	7 hr 36 min (247 min)	8 hr 11 min (255 min)
Perineal trauma (≥II), n (%)	8 (40)	10 (50)
Cesarean section, n (%)	10 (50)	9 (45)
Preterm birth, n (%)	2 (10)	2 (10)
Neonatal birth weight (g), mean (SD)	3286 (431)	3269 (449)
Low birth weight, n (%)	1 (5)	1 (5)
Neonatal asphyxia, n (%)	1 (5)	1 (5)
Macrosomia, n (%)	0 (0)	0 (0)
Congenital malformation, n (%)	0 (0)	0 (0)

For neonatal outcomes, mean birth weight was 3286 (SD 431) g in the intervention group and 3269 (SD 449) g in the control group. Low birth weight occurred in 1 out of 20 infants (5%) in each group, and neonatal asphyxia was also reported once in each arm. No macrosomia or congenital malformations were observed. Because these events were rare and the sample size was small, the absence of adverse neonatal outcomes should not be attributed to the intervention.

### Qualitative Results: Mechanisms of Change

Thematic analysis of postintervention interviews revealed 3 interrelated mechanisms of change through which the experiential class and online logging intervention shaped women’s experiences and practices: (1) empowerment and self-efficacy, (2) practice and persistence, and (3) systematic and environmental support.

#### Empowerment and Self-Efficacy Mechanism

Women consistently described gaining reassurance, confidence, and emotional support through the acquisition of knowledge, social interaction, and guidance from providers. These processes collectively enhanced their sense of self-efficacy. Clear explanations of physiological changes, delivery processes, and preparation for procedures reduced fear and built confidence:

*了解了这些知识之后，说实话就不是特别害怕了…了解了之后对生孩子还是挺有信心的。(After learning this knowledge, to be honest, I was no longer that afraid… once I understood what to expect, I felt much more confident about giving birth.)*

[WDK02]

Information about managing pregnancy risks also alleviated stress and created a sense of control:

*通过一日课程门诊，我就知道该如何去控制，然后心理压力就没那么大了。 (Through the one-day course clinic, I learned how to control it, and my psychological stress was much less.)*

[WDK01]

Beyond knowledge, women valued social interaction and trust in providers, which contributed to both informational and emotional empowerment:

*在群里面互动，互相学习，还有结交了几个比较好的朋友。 (In the group we interacted and learned from each other, and I even made a few good friends.)*

[DK08]

*首先信心上是给我们了一个很大的支持，因为我们知道只要遵医嘱就会有好的结果。 (First of all, it gave us a great deal of confidence, because we knew that as long as we followed the doctor’s advice, there would be good outcomes.)*

[DK19]

These accounts suggest that individual empowerment was reinforced by collective efficacy. Peer support and shared learning strengthened the women’s confidence to engage in healthy practices, laying the foundation for sustained practice change.

#### Practice and Persistence Mechanism

Building on enhanced self-efficacy, women described attempts to implement healthy practices, such as dietary adjustments, exercise, and blood glucose monitoring. Self-discipline and perseverance were recurrent themes, but the sources of motivation differed between groups. In the control group, women primarily relied on their own willpower:

*靠毅力…课程上他不给了那个那八段锦什么的，基本上一周我都会跳个两三次。 (I relied on willpower… in the class they gave us the Baduanjin exercises, and basically I practice them two or three times a week.)*

[WDK03]

In the intervention group, accountability and continuity through logging and interaction with providers or peers were emphasized:

*会有医护人员给我们监控，比如说我们吃了多少东西去打卡，什么时候该运动，什么时候该加餐…所以说这个特别好。 (Health care staff would monitor us-for example, what we logged about food intake, when we should exercise, when and how to add snacks. This was really very good.)*

[DK09]

*自己一个人有的时候确实难以坚持…有一个伙伴一起，这样比较容易坚持下来。 (On your own it’s really hard to keep going. Usually, you need a coach or a partner, that way it’s much easier to stick with it.)*

[DK03]

Some intervention participants further described how sustained practice became habitual, with long-term benefits that even carried over into subsequent pregnancies:

*比如现在我怀二胎…在抽静脉血的时候，那血糖都是一切都是正常的…不是有一种叫21天定律吗？坚持21天过后，然后你后面的你就形成了一种自然规律了。(For example, now I’m pregnant with my second child… when they tested my blood, everything was normal. There’s that saying about the 21-day rule-if you stick to something for 21 days, afterwards it becomes a natural routine.)*

[DK04]

We interpreted such statements as perceived trajectories of practice changes rather than verified long-term outcomes because the logging and quantitative assessment occurred only within the first week.

Women also noted reviewing their own past logging records to reinforce ongoing practices change:

*我会经常回顾之前孕期拍照打卡的那些照片…现在我的三餐它的搭配跟之前差多少？(I often look back at the photos I logged during pregnancy… and compare how my meals are balanced now versus back then.)*

[DK05]

These narratives suggest that participants perceived the logging component as increasing accountability and short-term persistence over the 7-day period. However, given the brief intervention and follow-up window, sustained practice maintenance or habit formation cannot be established in this study.

#### Systematic and Environmental Support Mechanism

Despite strong self-efficacy, participants emphasized that practice changes were shaped and sometimes constrained by systemic and environmental factors. Work schedules, family roles, and institutional arrangements acted as either facilitators or barriers. For instance, lack of time due to work hindered adherence:

*饮食时间不规律，下班太晚，晚饭吃得比较晚，然后也没时间运动，这是个瓶颈。 (My meal times are irregular-after work it’s too late, dinner ends up being very late, and then there’s no time to exercise. That’s a real bottleneck)*

[DK02]

On the other hand, adherence can also be influenced by family:

*家人做什么就吃什么…我家里人本身就比较注重这个养生这一边，所以就还是很好坚持的。 (I just eat whatever my family cooks… fortunately, they are very health-conscious, so it’s easy for me to stick with it.)*

[WDK04]

Institutional factors such as class timing also created participation barriers:

*有的时候主要是周六周日比较好一点，他就经常是在周二周四这种的，其实时间上有点不太恰当。 (It would be better on weekends, but the sessions were often on Tuesdays or Thursdays, which really wasn’t the most convenient.)*

[WDK07]

These findings illustrate that even when women were motivated and empowered, insufficient system-level support could undermine the sustainability of practices, whereas strong family and institutional backing enabled empowerment and practices to translate into lasting change.

## Discussion

### Principal Findings

This mixed methods study evaluated the feasibility and short-term, preliminary effects of integrating experiential antenatal classes with a 7-day digital logging component in a district-level maternal and child health institution setting in Beijing. We observed short-term improvements in maternal knowledge, selected self-reported practices, and service satisfaction, alongside underpowered and nonsignificant clinical trends (such as reduced labor duration and postpartum blood loss). Thematic analysis further illuminated a chain of mechanisms of change: self-efficacy was enhanced collectively by knowledge from experiential learning and interpersonal interaction and, in turn, translated into daily health practices under the influence of external supports or barriers. Together, these results provide new evidence that bridging participatory education with digital accompaniment can address persistent gaps in maternal health promotion.

### Comparison With Existing Evidence

The quantitative analysis demonstrated a significant incremental effect provided by experiential classes and online logging on the knowledge and practices of participants in the intervention group. The further enhancement of these 2 indicators could be associated with each other. Knowledge gains are driven by feedback loops enabled by online logging practices. For example, women posting photos of meals or asking doctors questions via WeChat allow doctors or peers to correct misconceptions or address individual gaps. This is consistent with recent literature. For instance, He et al [32] found that WeChat‐based prenatal education’s real-time communication between patients and clinicians enables clarification of misunderstandings and strengthens comprehension. Wu et al [33] demonstrated that caregiver feeding knowledge was improved not just through message delivery but through caregiver queries and interactions in online groups. Furthermore, regular practices facilitated by online logging can be seen as an extension of experiential learning. Concrete action and reflection can deepen understanding and embed skills in daily routines, helping knowledge “stick” in practice [16].

The intervention group’s superior behavioral outcomes suggest that the digital logging component reinforced continuity of support. However, the results from similar digital interventions are mixed: some trials show strong improvements in adherence and dietary or gestational weight outcomes, whereas others report minimal or no practice changes beyond standard care [34-36]. Our qualitative findings suggest another contributing mechanism: digital logging made shared goals more explicit, and therefore, participants reported feeling socially motivated. It aligns with the concept of collective efficacy, where group norms and peer visibility contribute to sustained practices. Studies in participatory interventions show that when group members believe in shared capability and observe others’ progress, adherence is more likely [37,38].

Although differing in scale, participants in both control and intervention groups reported that gaining knowledge through antenatal education helped reduce nervousness and fear. In our quantitative results, both groups rated high satisfaction with services. This finding resonates with other studies of interactive or group-based antenatal education (small classes, discussions, etc), such as Augar et al [39] and Brixbal et al [40]. There is also evidence that good satisfaction contributes to continuity of care and positive health outcomes [41,42]. These mechanisms are consistent with contemporary ANC guidance that places women’s experience, communication quality, and continuity of support at the center of care [11].

Additionally, participants in our study reported that both external and environmental conditions inhibited and facilitated practice change. This is consistent with qualitative evidence. For example, Escañuela Sánchez et al [43] show that even when women have awareness and motivation, social norms, stigma, and poor interaction with health care professionals often block effective weight management practices. Similarly, Jhaveri et al [44] in Uttar Pradesh find that structural opportunity barriers, such as lack of transport, limited food availability, and financial constraints, prevent pregnant women from translating knowledge into consistent nutrition, ANC attendance, or supplement intake. These findings reinforce that didactic changes (knowledge alone) are insufficient and practice changes depend critically on environmental support and opportunity.

### Implications for Practice and Policy

The convergence of quantitative improvements and qualitative insights underscores the feasibility of scaling this model in urban ANC settings. The intervention required minimal additional resources beyond routine classes, leveraging an existing and widely adopted digital platform (WeChat) for continuity of care. As China continues to prioritize maternal and child health under the Healthy China 2030 strategy, integrating participatory education with digital reinforcement could serve as a cost-effective approach to optimize antenatal services, particularly in tertiary hospitals where patient volume and complexity are high. Furthermore, the qualitative findings identified emotional reassurance, trust, and peer connection as important dimensions of care that are not adequately captured by traditional clinical indicators but are essential for improving service quality and women’s lived experiences. However, we must acknowledge that scalability may differ substantially outside urban district hospitals. In rural or low-connectivity areas, limited internet access, lower smartphone penetration, and constrained staffing may reduce feasibility or require alternative modalities (eg, SMS text messaging–based prompts, offline-capable tools, or community health worker support). Implementation should be adapted to local digital infrastructure and population needs rather than assumed transferable from an urban, digitally literate sample.

### Strengths and Limitations

To our knowledge, this is the first study integrating experiential antenatal classes with digital logging in maternal health promotion. The mixed methods design not only allowed for outcome measurement but also provided explanatory insights into mechanisms of change. By situating the intervention in a district-level maternal and child health hospital rather than a tertiary setting, the study reflects the real-world context where most women receive ANC, thereby enhancing external validity. The intervention itself was low cost and built on existing tools such as WeChat, making it adaptable and scalable in diverse settings. Moreover, outcomes were assessed across multiple dimensions, including knowledge, practices, satisfaction, and clinical indicators, broadening the evaluation scope and practical relevance of findings.

Nonetheless, limitations should be acknowledged. The modest sample size and quasi-experimental design without randomization limit statistical power and raise the possibility of residual confounding. In addition, knowledge, practices, and satisfaction were assessed only once post intervention, and the digital logging component was restricted to a short 7-day period, leaving the long-term sustainability uncertain. Furthermore, implementation relied on clinicians’ voluntary after-hours engagement to provide feedback, which may affect scalability under routine service conditions. When scaled, this model may require explicit workflow design (eg, protected time, standardized feedback templates, tiered escalation rules, or task-shifting to nurses/health educators) to avoid increasing clinician burden. Semiautomated reminders and rule-based feedback could preserve responsiveness while reducing manual workload but should be evaluated for safety and acceptability. Finally, participants were recruited from a single district-level hospital in Beijing, which may limit generalizability to other regions or lower-level facilities.

In addition, internal validity threats should be considered. First, the nonrandomized allocation and the small sample size increase the risk of selection bias and residual confounding (eg, baseline motivation, family support, and prior digital health engagement). Second, contamination is possible if participants shared information across groups. Third, key behavior outcomes relied on self-report and may be influenced by social desirability or Hawthorne effects. Future studies should use randomized (or cluster-randomized) designs with allocation concealment where feasible, incorporate objective behavioral indicators (eg, wearable activity data or clinic-verified glucose logs), and include longer follow-up periods to assess maintenance.

Equity considerations are also important. The digital component may preferentially benefit women with higher digital literacy, stable smartphone access, and supportive household environments. Women with limited autonomy, heavy caregiving/work burdens, or privacy concerns may be less able to log consistently or engage in provider messaging. Future implementation should include equity-oriented adaptations (eg, simplified logging, optional voice input, family-inclusive education when appropriate, and alternative low-tech reinforcement pathways).

### Future Directions

Future studies can test longer logging periods (eg, 4‐12 wk) and/or periodic “booster” check-ins (eg, weekly prompts or trimester-based logging windows) to evaluate maintenance, burden, and engagement trajectories over time. Future research should evaluate this combined model in larger, multisite randomized controlled trials with longer-term follow-up to determine the sustainability of effects. Incorporating objective measures of practice changes, such as wearable physical activity trackers or biochemical indicators, would enhance validity beyond self-reported outcomes. Given that external and family environments strongly influence maternal practices, future studies should also explore family-inclusive education models. Moreover, investigating adaptation in resource-constrained settings will be essential for assessing scalability and equity. Finally, research into the mechanisms behind digital logging, such as accountability, social motivation, and feedback loops, could inform the design of more targeted and effective digital components.

### Conclusion

This study provides preliminary evidence that integrating experiential antenatal classes with digital logging is a feasible, acceptable, and potentially effective model for promoting maternal health practices in China. The intervention improved knowledge, adherence, and satisfaction, with qualitative insights clarifying mechanisms of practice changes. While further research with larger samples is needed, the combined experiential-digital model offers a promising pathway for bridging the gap between knowledge and sustained health practices in ANC.

## Abbreviations

ANC: antenatal care
WHO: World Health Organization

## References

[1] Say L, Chou D, Gemmill A (2014). Global causes of maternal death: a WHO systematic analysis. Lancet Glob Health.

[2] Neiger R (2017). Long-term effects of pregnancy complications on maternal health: a review. J Clin Med.

[3] (2019). Maternal mortality: levels and trends 2000 to 2017. https://www.un.org/development/desa/pd/sites/www.un.org.development.desa.pd/files/unpd_unicef_level_and_trends_in_maternal_mortality_2000-2017-eng.pdf.

[4] Tan X, Liu X, Shao H (2017). Healthy China 2030: a vision for health care. Value Health Reg Issues.

[5] Tang S, Meng Q, Chen L, Bekedam H, Evans T, Whitehead M (2008). Tackling the challenges to health equity in China. Lancet.

[6] Amiri FN, Faramarzi M, Bakhtiari A, Omidvar S (2021). Risk factors for gestational diabetes mellitus: a case-control study. Am J Lifestyle Med.

[7] Leng J, Shao P, Zhang C (2015). Prevalence of gestational diabetes mellitus and its risk factors in Chinese pregnant women: a prospective population-based study in Tianjin, China. PLoS One.

[8] Alder J, Fink N, Bitzer J, Hösli I, Holzgreve W (2007). Depression and anxiety during pregnancy: a risk factor for obstetric, fetal and neonatal outcome? A critical review of the literature. J Matern Fetal Neonatal Med.

[9] Dunkel Schetter C, Tanner L (2012). Anxiety, depression and stress in pregnancy: implications for mothers, children, research, and practice. Curr Opin Psychiatry.

[10] Muktabhant B, Lawrie TA, Lumbiganon P, Laopaiboon M (2015). Diet or exercise, or both, for preventing excessive weight gain in pregnancy. Cochrane Database Syst Rev.

[11] (2016). WHO recommendations on antenatal care for a positive pregnancy experience. https://iris.who.int/server/api/core/bitstreams/9dccde13-3593-4a22-9237-61abe5a3c6b7/content.

[12] Walker RE, Choi TST, Quong S, Hodges R, Truby H, Kumar A (2020). “It’s not easy” - a qualitative study of lifestyle change during pregnancy. Women Birth.

[13] Xue W, Cheng KK, Liu L (2023). Barriers and facilitators for referring women with positive perinatal depression screening results in China: a qualitative study. BMC Pregnancy Childbirth.

[14] Xu Y, Tang Y, Wang M (2025). Midwives perspectives of barriers and facilitators for the practice of promoting women’s positive childbirth experience in China: a qualitative study. Women Birth.

[15] Billett S (2016). Learning through health care work: premises, contributions and practices. Med Educ.

[16] Kolb DA (2014). Experiential Learning Experience as the Source of Learning and Development.

[17] Yardley S, Teunissen PW, Dornan T (2012). Experiential learning: transforming theory into practice. Med Teach.

[18] Bandura A (1997). Self-Efficacy The Exercise of Control.

[19] Olander EK, Atkinson L, Edmunds JK, French DP (2011). The views of pre- and post-natal women and health professionals regarding gestational weight gain: an exploratory study. Sex Reprod Healthc.

[20] Chan HHK, Kwong HYC, Shu GLF, Ting CY, Lai FHY (2021). Effects of experiential learning programmes on adolescent prosocial behaviour, empathy, and subjective well-being: a systematic review and meta-analysis. Front Psychol.

[21] Roche D, Rafferty A, Holden S, Killeen SL, Kennelly M, McAuliffe FM (2022). Maternal well-being and stage of behaviour change during pregnancy: a secondary analysis of the PEARS randomised controlled trial. Int J Environ Res Public Health.

[22] Zinsser LA, Stoll K, Wieber F, Pehlke-Milde J, Gross MM (2020). Changing behaviour in pregnant women: a scoping review. Midwifery.

[23] Marcolino MS, Oliveira JAQ, D’Agostino M, Ribeiro AL, Alkmim MBM, Novillo-Ortiz D (2018). The impact of mHealth interventions: systematic review of systematic reviews. JMIR mHealth uHealth.

[24] Chen H, Chai Y, Dong L, Niu W, Zhang P (2018). Effectiveness and appropriateness of mHealth interventions for maternal and child health: systematic review. JMIR mHealth uHealth.

[25] Luan H, Wang M, Sokol RL, Wu S, Victor BG, Perron BE (2020). A scoping review of WeChat to facilitate professional healthcare education in Mainland China. Med Educ Online.

[26] McCowan LME, Dekker GA, Chan E (2009). Spontaneous preterm birth and small for gestational age infants in women who stop smoking early in pregnancy: prospective cohort study. BMJ.

[27] Lee SH, Nurmatov UB, Nwaru BI, Mukherjee M, Grant L, Pagliari C (2016). Effectiveness of mHealth interventions for maternal, newborn and child health in low- and middle-income countries: systematic review and meta-analysis. J Glob Health.

[28] Creswell JW, Clark VLP (2017). Designing and Conducting Mixed Methods Research.

[29] Launiala A (2009). How much can a KAP survey tell us about people’s knowledge, attitudes and practices? Some observations from medical anthropology research on malaria in pregnancy in Malawi. Anthropol Matters.

[30] Altman DG (1990). Practical Statistics for Medical Research.

[31] Braun V, Clarke V (2006). Using thematic analysis in psychology. Qual Res Psychol.

[32] He Y, Fan G, Fan G, Liu D (2025). Exploring nurse and patient perspectives on WeChat-based prenatal education in Chinese public hospitals: a qualitative inquiry. BMC Nurs.

[33] Wu Q, Huang Y, Helena van Velthoven M, Wang W, Chang S, Zhang Y (2021). Feasibility of using WeChat to improve infant and young child feeding in rural areas in China: a mixed quantitative and qualitative study. PLoS One.

[34] Jaeger KM, Nissen M, Leutheuser H (2025). Adherence to digital pregnancy care - lessons learned from the SMART start feasibility study. NPJ Digit Med.

[35] Wei HX, Yang YL, Luo TY, Chen WQ (2023). Effectiveness of mobile health interventions for pregnant women with gestational diabetes mellitus: a systematic review and meta-analysis. J Obstet Gynaecol.

[36] Alnughaymishi R, Alsaeed N, Alharbi R (2024). The impact of digital interventions and social media on maternal health amongst pregnant women and new mothers: a systematic review and meta-analysis of randomised controlled trials. J Adv Trends Med Res.

[37] Kuchenbaur M, Peter R (2021). Assessing the role of collective efficacy beliefs during participative occupational health interventions. Front Public Health.

[38] Butel J, Braun KL (2019). The role of collective efficacy in reducing health disparities: a systematic review. Fam Community Health.

[39] Auger SJ, Verbiest S, Spickard JV, Simán FM, Colindres M (2015). Participatory group prenatal education using photonovels: evaluation of a lay health educator model with low-income Latinas. J Particip Med.

[40] Brixval CS, Axelsen SF, Thygesen LC, Due P, Koushede V (2016). Antenatal education in small classes may increase childbirth self-efficacy: results from a Danish randomised trial. Sex Reprod Healthc.

[41] Galle A, Van Parys AS, Roelens K, Keygnaert I (2015). Expectations and satisfaction with antenatal care among pregnant women with a focus on vulnerable groups: a descriptive study in Ghent. BMC Womens Health.

[42] Yussuf NIH, Mahande MJ, Manongi RN, Stekelenburg J (2025). Level of satisfaction with the quality of antenatal care services in public health facilities and associated factors among pregnant women in Unguja, Zanzibar. BMC Pregnancy Childbirth.

[43] Escañuela Sánchez T, Meaney S, O’Connor C (2022). Facilitators and barriers influencing weight management behaviours during pregnancy: a meta-synthesis of qualitative research. BMC Pregnancy Childbirth.

[44] Jhaveri NR, Poveda NE, Kachwaha S, Comeau DL, Nguyen PH, Young MF (2023). Opportunities and barriers for maternal nutrition behavior change: an in-depth qualitative analysis of pregnant women and their families in Uttar Pradesh, India. Front Nutr.

